# Temperature-Dependent Selectivity and Detection of Hidden Carbon Deposition in Methane Oxidation

**DOI:** 10.1021/acscatal.4c00228

**Published:** 2024-04-05

**Authors:** Ulrike Küst, Weijia Wang, Changda Wang, Helena Hagelin-Weaver, Johan Gustafson, Andrey Shavorskiy, Jason F. Weaver, Jan Knudsen

**Affiliations:** † Division of Synchrotron Radiation Research, Lund University, P.O. Box 118, SE-221 00 Lund, Sweden; ‡ NanoLund, Lund University, P.O. Box 118, SE-221 00 Lund, Sweden; § MAX IV Laboratory, Lund University, P.O. Box 118, SE-221 00 Lund, Sweden; ∥ National Synchrotron Radiation Laboratory, University of Science and Technology of China, Hefei 230029, China; ⊥ Department of Chemical Engineering, University of Florida, Gainesville, Florida 32611, United States

**Keywords:** methane oxidation, palladium, APXPS, carbon deposition, time-resolved

## Abstract

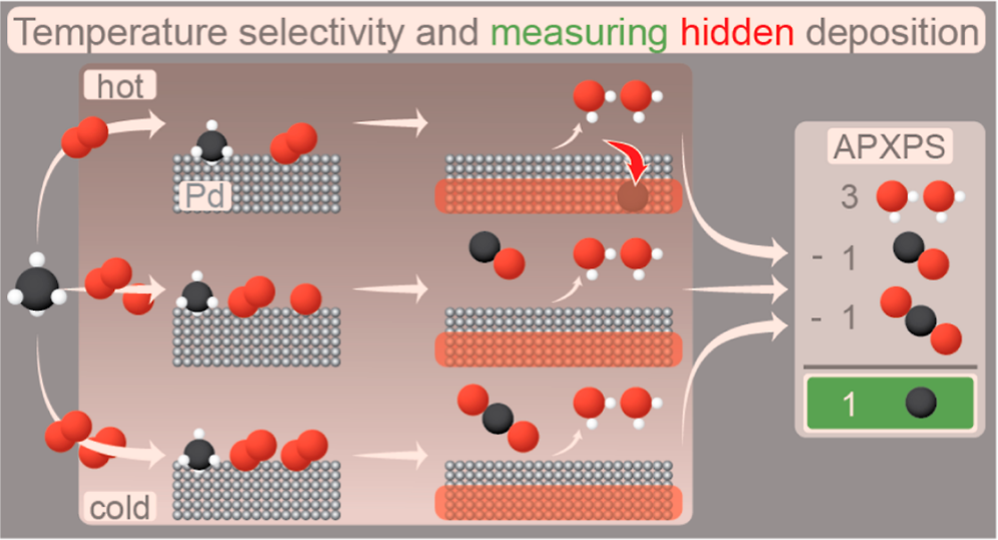

Reaction products in heterogeneous catalysis can be detected either on the catalyst surface or in the gas phase after desorption. However, if atoms are dissolved in the catalyst bulk, then reaction channels can become hidden. This is the case if the dissolution rate of the deposits is faster than their formation rate. This might lead to the underestimation or even overlooking of reaction channels such as, e.g., carbon deposition during hydrocarbon oxidation reactions, which is problematic as carbon can have a significant influence on the catalytic activity. Here, we demonstrate how such hidden deposition channels can be uncovered by carefully measuring the product formation rates in the local gas phase just above the catalyst surface with time-resolved ambient pressure X-ray photoelectron spectroscopy. As a case study, we investigate methane oxidation on a polycrystalline Pd catalyst in an oxygen-lean environment at a few millibar pressure. By ramping the temperature between 350 and 525 °C, we follow the time evolution of the different reaction pathways. Only in the oxygen mass-transfer limit do we observe CO production, while our data suggests that carbon deposition also happens outside this limit.

## Introduction

Carbon deposition is often observed during hydrocarbon reactions under oxygen-lean conditions. If the deposited carbon accumulates on the surface, it is detectable with standard surface-sensitive techniques used to study heterogeneous catalysis. However, it can become hidden when dissolution in the bulk is faster than deposition itself. Hence, the amount of carbon deposition is underestimated or even overlooked.

Carbon deposition is not only an illustrative example of a deposition reaction; it can also have a significant impact on the catalytic activity.^1−3^ However, it is still debated whether it is a spectator, inhibits, or promotes the reaction. An inhibiting effect of carbon has, for example, been reported by refs (4) and (5), while more recent dry reforming methane studies reported no deactivating effect of carbon.^3^ More recent work found that carbon has a promotional effect on catalytic activity,^1,2,6,7^ and studies on CO_2_ electroreduction on copper-based catalysts even found that the nature of carbon influences the reaction product.^8^

In the present work, a polycrystalline palladium surface is chosen as a catalyst since it is a good oxidation catalyst for methane.^9,10^ For palladium, it is known that CO_2_ production dominates when oxygen is abundant,^11^ while CO production is only observed when O_2_ is the limiting reactant for the oxidation reaction.^12^ The transition from a catalyst surface that produces CO_2_ to one producing CO has, however, not been studied in detail before. We do this in the present work by gradually changing the selectivity from CO_2_ toward CO production while following both the surface and local gas composition with time-resolved ambient pressure X-ray photoelectron spectroscopy (tr-APXPS). The gradual transition is achieved by ramping the temperature in oxygen-lean conditions. Hereby—and according to the Arrhenius equation—the increased catalyst temperature leads to an increase in the reaction rate. As a result, the local gas composition in the vicinity of the surface becomes increasingly depleted of O_2_. This eventually moves the reaction into an oxygen mass-transfer limit (O-MTL) regime, in which the reaction rate is limited by the mass transport of oxygen through the gas phase to the surface. Once in the O-MTL, we observed CO formation. Our study is thus different from most published methane oxidation studies, which have been conducted in oxygen-rich environments.^9,10^

Before we discuss methane oxidation in more detail, it is instructive to compare it with the much simpler CO oxidation reaction and, in particular, what happens to this reaction once it becomes oxygen mass transfer limited. Opposite to methane oxidation, only one possible reaction pathway exists for CO oxidation, that is the oxidation to CO_2_. If this reaction is mass transfer limited by oxygen, the total CO conversion stagnates and the local partial pressure of CO increases at the surface, which eventually results in CO poisoning of the catalyst surface.^13−15^ In contrast, methane oxidation has three major oxidation pathways; thus, its behavior in the O-MTL is more complicated. Here, we consider only those reaction pathways for which we have experimental evidence, even though many others exist, such as those forming hydrogen, methanol, formaldehyde, or formic acid. The term methane oxidation will in the present article be used for any reaction involving the oxidation of any part of the dissociated methane. Other than carbon deposition
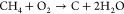
1these oxidation pathways are complete oxidation
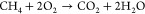
2and partial oxidation
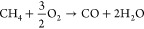
3

Opposite to the CO oxidation reaction, where the only solution to a limited oxygen supply is stagnation in CO_2_ production, the palladium surface can maintain or even increase the turnover of CH_4_ in the O-MTL either by opening up the carbon deposition or the partial oxidation reaction channels. What happens in detail when methane oxidation enters and exits the O-MTL is, however, not known, and many scientific questions are at present unanswered. To mention a few, it is unknown whether the CH_4_ turnover in the O-MTL increases or not. It is also unknown if either the partial oxidation or the carbon deposition reaction channel opens first or whether they open simultaneously. Finally, the correlation between the surface structure and partial oxidation is unknown. Ultimately, one needs to follow the evolution of the local gas composition, carbon, both deposited onto the surface and diffusing into the bulk, as well as the surface structure with high time resolution while driving the reaction into and out of the O-MTL. Using temperature ramps, this is exactly what we do in the present article. We find an increased CH_4_ turnover in the O-MTL due to both rapid carbon diffusion into the bulk and CO production. Surprisingly, we also observe carbon deposition outside the O-MTL.

## Experimental Section

The experiments presented here were performed at the HIPPIE beamline at the MAX IV laboratory in Lund, Sweden.^16^ Two different XPS measurement positions were used in the experiment: the “normal” XPS position found by maximizing the surface signal (≈300 μm distance between sample and 300 μm aperture of the cone used to collect the photoelectron) and the gas-phase measurement position where the sample is retracted by 400 μm, thereby measuring a gas-phase signal with little surface background. The photon energies for the different core levels were chosen, aiming for maximum surface sensitivity and a small background induced by the analyzer transmission function. The sample preparation is described in detail in the Supporting Information.

In the methane-rich gas environments that are necessary to observe carbon deposition, continuous carbon deposition and removal reportedly leads to surface roughening and deterioration of single crystals.^17−20^ For the present work, we used a polycrystalline surface obtained from a Pd(100) single crystal by continuous carbon deposition and removal. In this crystal, an almost infinite amount of bulk material is available for carbon diffusion, i.e., continuous carbon removal from the surface, in contrast to nanoparticles which are the usually chosen alternative^9,10,21^ in fuel-rich environments. This might change the observed catalytic behavior.

**Figure 1:**
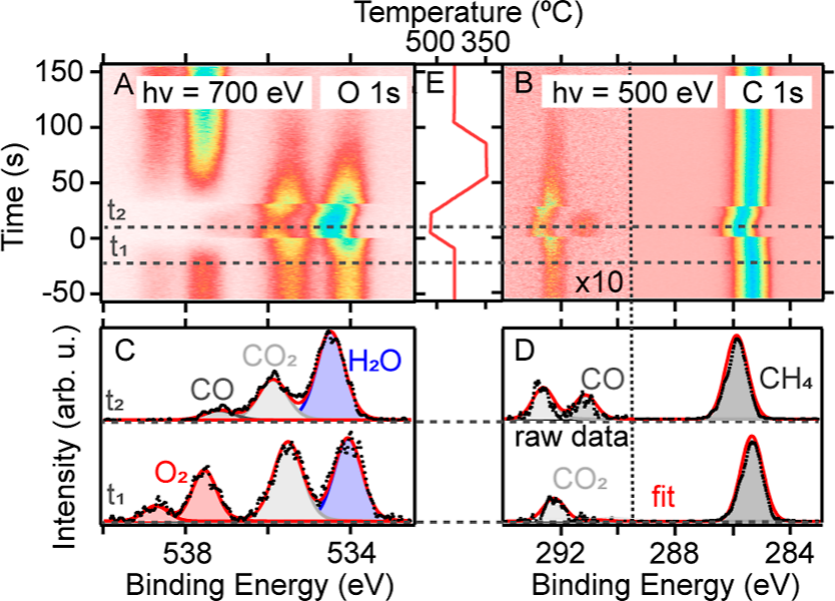
Measured raw data, i.e., the temperature of the catalyst, O 1s (A) and C 1s (B) gas-phase spectra. The left side of the C 1s spectra is multiplied by a factor of 10. Example of fit to the raw data for two different times, displaying the individual molecular contributions in C,D.

Here, we exposed the catalyst to temperature ramps in a methane-rich gas environment at a total pressure of 3.8 ± 0.3 mbar. The gas flows were 3.5 sccm CH_4_ and 0.5 sccm O_2_, while the temperature was ramped from 450 to 525 °C with a heating rate of 5 °C/s. After a dwell time of 30 s, the catalyst was cooled to 350 °C and, another 60 s later, heated to 450 °C again. The sample was kept at 450 °C for 900 s to allow for sufficient oxidation. Of the whole temperature cycle, however, only the time region around the O-MTL (which can be seen in Figure 1E) is discussed. That is, only the temperature ramps and the first 70 s of 900 s at 450 °C are presented. In this environment, the catalyst surface switched back and forth between oxide and carbide coverage. The surface was exposed to three consecutive temperature ramps to ensure reproducibility (Figure S1), while APXPS spectra were measured continuously with an acquisition frequency of 5 Hz. More details of the experimental setup can be found in Supporting Information, together with a discussion about gas diffusion limits in this cell setup.

## Results and Discussion

### Gas-Phase Spectra

O 1s and C 1s gas-phase tr-APXPS data, recorded in the gas-phase measurement position, are shown in Figure 1A,B, together with the catalyst temperature for the time around the O-MTL. Panels C and D show examples of the fitting at two times, *t*_1_ (at 450 °C) and *t*_2_ (at 525 °C), as indicated in panels A and B. For better visibility, the area around the CO and CO_2_ components in the C 1s spectra was multiplied by a factor of 10 in panels B and D. For time-alignment, *t* = 0 was chosen as the time at which all gas-phase components experience a shift of +0.4 eV in their apparent binding energy (BE). This common behavior is due to a change in the sample work function, i.e., a change in the chemical state of the surface.

Discussing the most prominent results in Figure 1, we observe major changes in the catalytic activity. First, the temperature ramp brings the catalyst into and out of the oxygen mass-transfer limit (O-MTL) since the oxygen signal drops to zero at time values near *t*_2_. CH_4_, instead, is detected during the entire measurement. During the O-MTL, we observe that CO is being produced, and the intensity of the CO_2_ signal reduces significantly. Additionally, shifts in the apparent BE of all gas-phase components, i.e., a work function shift of the sample, are observed. This seems to be correlated with the absence and presence of gaseous oxygen, respectively.

**Figure 2:**
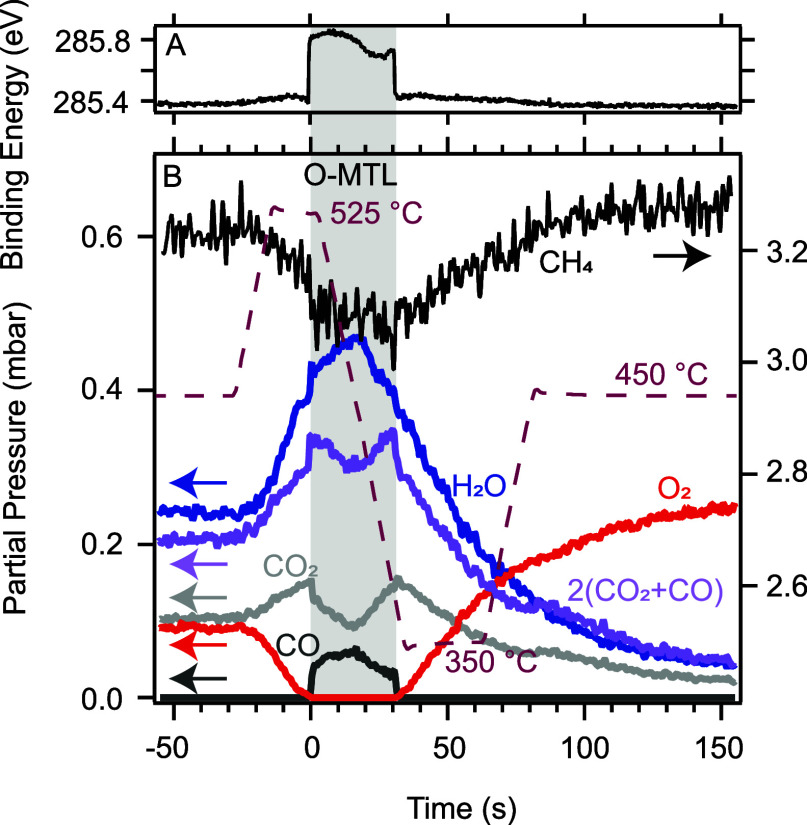
Apparent C 1s binding energy fit results for methane are shown (A). Partial pressure fit results to the C 1s (CH_4_) and the O 1s (H_2_O, CO_2_, CO) data are shown in panel B together with twice the sum of the CO and CO_2_ partial pressures for later analysis. The methane component is accounted for by the right-hand axis while the other components refer to the left one. The temperature of the catalyst is shown with a dashed line.

To determine the nature of this correlation in more detail, the data in Figure 1A,B are fitted, resulting in an apparent BE of the components as shown in Table 1 at times *t*_1_ and *t*_2_. To estimate partial pressures, a scaling factor was first applied to the curve-fitted intensities to account for different photoionization cross sections of O 1s and C 1s peaks. Partial pressures were then computed from the scaled peak intensities by dividing by the number of corresponding atoms in the probed molecule and scaling to the measured total pressure. The obtained partial pressures are binomially smoothed 75 times and presented in Figure 2 with the resulting error bars shown in Figure S2. For the time development of CO and CO_2_, only the O 1s fit results are shown since the C 1s results are similar (see Figure S2). Only the apparent C 1s BE of gas-phase methane is shown, since the other components display the same behavior (see Figure S1A).

**Table 1:**
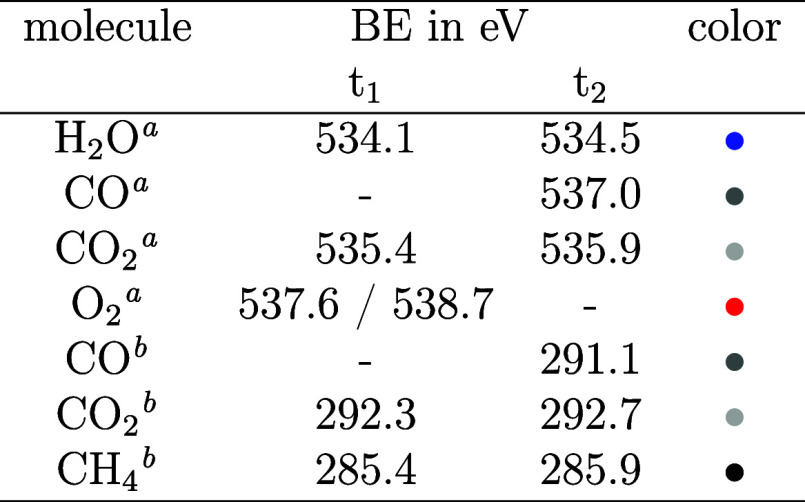
Apparent Binding Energies of All Gas-Phase Components Obtained from the Fit to the Spectra

a Measured in O 1s.

b Measured in C 1s.

At the beginning of the temperature ramp, i.e., at *t* < 0 s, the catalyst activity must limit the reaction since both O_2_ and CH_4_ are observed in the gas phase, as visible in Figure 2B. Subsequently, at *t* = 0 s, after the catalyst temperature has resided at 525 °C for approximately 15 s, the O_2_ pressure above the surface drops to zero. From this point onward, the conversion rate is limited by the diffusion of oxygen to the surface. This limitation remains until ≈30 s, when the catalyst temperature almost reaches 350 °C and an oxygen gas-phase signal is measured again. From here on, the surface activity limits the conversion rate again. Therefore, the reaction in the time interval from 0 to 30 s is in the O-MTL, as indicated by the gray shaded area. This, however, does not mean that no oxygen reaches the surface in the O-MTL, but rather that all oxygen that does reach the surface is immediately consumed.

During the O-MTL, an insufficient oxygen supply leads to an interesting effect. As opposed to CO oxidation, in which mass-transfer limitations result in a stagnating conversion rate with no changes in the reaction product composition, for methane oxidation, changes in the product composition are observed. Indeed, it can be seen in Figure 2B that CO is only observed in the O-MTL, i.e., between *t* = 0 and *t* ≈ 30 s. This is sensible since having an oxygen mass-transfer-limited reaction in the case of methane oxidation means that that reaction pathway will shift toward those oxidation reactions that consume less oxygen. Thus, observing a reduced CO_2_ partial pressure during the O-MTL is reasonable according to the same argumentation.

Discussing the apparent C 1s BE of methane in Figure 2A, it can be seen that the O-MTL also affects the catalyst surface phase. The sudden changes in the apparent BE at *t* = 0 and *t* = 30 s are caused by a sample work function change, which indicates rapid chemical transitions on the surface. Here, the work function changes between two consecutive measurement points in the unsmoothed data (see Figure S2), which points toward a surface development faster than the sampling rate, i.e., 0.2 s. Since these rapid changes occur simultaneously with entering into and exiting the O-MTL, an indication is provided that the absence of oxygen in front of the sample triggers a change in the surface phase.

Analyzing the balanced reaction equations in more detail (eqs 1 to 3), it can be seen that every converted CH_4_ molecule must result in two H_2_O molecules. Thus, the water pressure can be taken as a measure of the total methane conversion rate. Even if hydrogen is produced (eq S1), the water pressure can still be taken as a measure for the minimum conversion rate of methane. In the following analysis, we treat the water pressure as a measure of the total conversion rate since hydrogen formation is assumed to be a minority reaction channel in the presence of oxygen. This even has advantages over taking the measured methane signal as an indicator since the initial methane pressure in front of the sample, without any conversion, is not known. The reaction balance then leads to the conclusion that the H_2_O pressure must be equal to twice the sum of the CO_2_ and CO pressures if no carbon is formed on the surface. When examining Figure 2, it can be seen that while the H_2_O pressure is indeed equal to 2 × (CO_2_ + CO) (purple curve) for >80 s, this is not the case for the remaining presented time region. Here, the deviation between H_2_O and 2 × (CO_2_ + CO) is significant within the O-MTL, i.e., for 0 < *t* < 30 s and minor outside of it, i.e., for −55 s < *t* < 0 and 30 s < *t* < 80 s. This is an indication that carbon is being deposited on the surface already shortly before, during, and shortly after the O-MTL.

When estimating the amount of deposited carbon during the O-MTL, we take the average partial pressure of water resulting from carbon deposition in that time range. That is, the average difference between the total water signal and 2 × (CO_2_ + CO), which is 0.1 mbar for 0 < *t* < 30 s. Thus, roughly 0.05 mbar of CH_4_ is converted to deposited carbon. This is equivalent to the formation of roughly 2 × 10^4^ monolayers (ML) of carbon every second, according to the kinetic gas theory^22^ (see Supporting Information for detailed calculation). Within 30 s of the formation of the O-MTL, this corresponds to the formation of approximately 6 × 10^5^ ML.

Now the evolution of the methane conversion probed by the water pressure evolution is discussed. The maximum conversion rate is found approximately in the middle of the O-MTL at *t* = 18 s. At the same time, the CO pressure is at its maximum, and the CO_2_ pressure reaches a minimum value in the O-MTL region, which is reasonable because the maximum conversion rate occurs while the oxygen supply is limited. Meanwhile, a slight drop in the apparent C 1s BE of methane indicates a changing surface phase. After exiting the O-MTL, the conversion rate, i.e., the H_2_O pressure, drops until the catalyst becomes almost inactive. This is a very intriguing observation; even though the sample temperature is at 450 °C three times during one cycle, i.e., *t* < −30 s, *t* ≈ 15 s, and *t* > 80 s, the conversion rate is dramatically different at these times. This fact already excludes an explanation for the changed activity due to temperature according to the Arrhenius equation. Instead, different surface phases must be responsible for the changes in activity.

When analyzing the development of the apparent C 1s BE of methane in Figure 2A, not only the rapid changes at the beginning and end of the O-MTL, that were already discussed, can be observed. Additionally, in the middle of the O-MTL, i.e., at *t* ≈ 15 s, the work function shifts again, which indicates a changing surface phase even within the O-MTL, while the conversion rate simultaneously reaches a maximum. But also, outside of the O-MTL, a continuous increase in the apparent BE for *t* < 0 and a gradual decrease for *t* > 30 s is observed. This is an indication of a continuously changing surface phase of the catalyst even outside of the O-MTL.

To summarize, the most important conclusion obtained in this section is that the methane conversion and the formation of different reaction products also change within the O-MTL as opposed to those of CO oxidation. This conclusion follows directly from the gas-phase spectra of the O 1s and C 1s and from realizing that the water pressure is a measure for the (minimum) methane conversion. In more detail, the methane conversion increases in the first half of the O-MTL, shifting the reaction pathway selectivity away from complete oxidation (CO_2_ formation) in favor of partial oxidation (CO production) and carbon deposition, even though all three reaction pathways are still present. This results in a peaking conversion rate in the middle of the O-MTL. Surprisingly, a minor carbon deposition is observed shortly before and after the O-MTL. Finally, it is clear that CO formation is observed solely during the O-MTL.

### Surface Spectra

**Figure 3:**
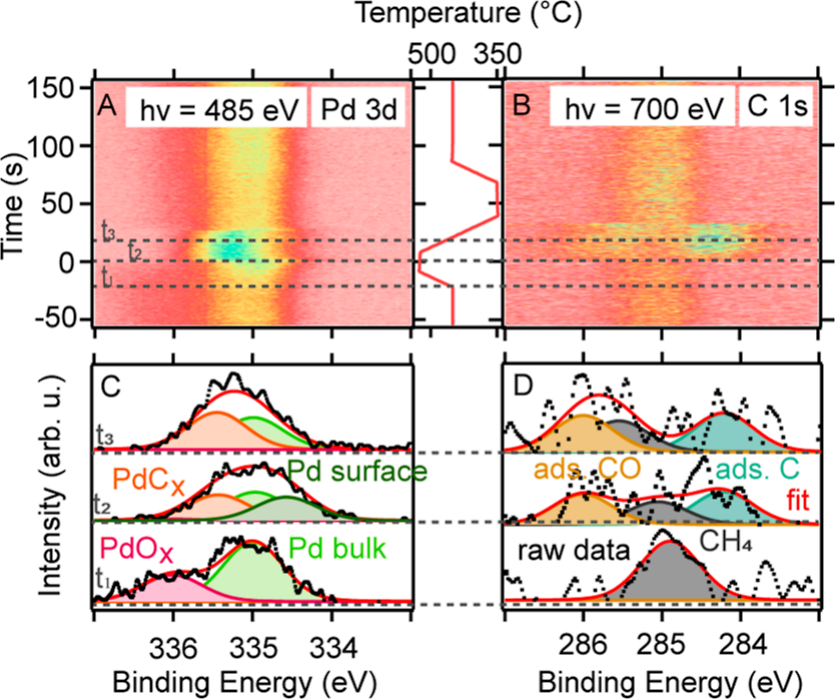
Measured raw data, i.e., the temperature of the catalyst, Pd 3d_5/2_ (A) and C 1s (B) surface spectra. Examples of fit to the raw data in panels (C, D).

In Figure 3, the Pd 3d_5/2_ (A) and C 1s (B) surface spectra are shown together with the sample temperature for a limited time around the O-MTL. Also, the O 1s spectra were measured, but since the most interesting changes occur in the Pd 3d_5/2_ and C 1s spectra, the O 1s results are only presented in Figure S3. All spectra were recorded under experimental conditions identical with the gas-phase spectra discussed above. Panels C and D show examples of the fit to the measured spectra using binding energies as documented in Table 2 (averaged over 10 spectra in the time-direction) at three times, *t*_1_, *t*_2_, and *t*_3_, as indicated in panels A and B. When fitting the C 1s spectra, we chose to include two components in the peak in the raw data centered at 285.9 eV. The work function shift, which applies to the methane apparent C 1s BE, is known from the gas-phase discussion (+0.4 eV). If that shift is applied to the methane apparent C 1s BE measured here (285.1 eV), the peak at 285.9 eV cannot be fitted with only the CH_4_ component. Also, since we produce gaseous CO, a respective surface component of adsorbed CO is expected at 286.0 eV.^21^ Combining two peaks at the respective expected BEs results in a good fit to the raw data, as shown in Figure 3D. For the Pd 3d_5/2_ spectra, four components are identified: the Pd bulk contribution, a component of the metallic surface, one of the oxide, and one of the carbide. As the exact composition of the palladium oxide and carbide phases is difficult to determine, the phases are referred to as PdO_*x*_ and PdC_*x*_ hereafter. For PdC_*x*_, that includes even the Pd atoms bound to adsorbed CO.

**Table 2:**
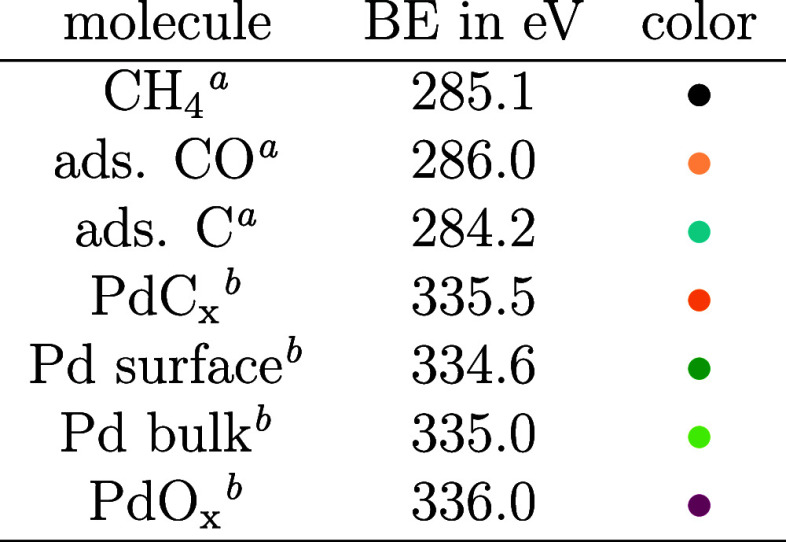
Binding Energies of All Surface Components Obtained from the Fit to the Spectra

a Measured in C 1s.

b Measured in Pd 3d_5/2_.

The key observations in Figure 3 are that the oxide (PdO_*x*_) vanishes during the O-MTL (0 < *t* < 30 s) while a carbide phase (ads. CO, ads. C, and PdC_*x*_) is detected on the surface. In the Pd 3d_5/2_ spectra (panels A and C), it becomes visible that the removal of the oxide (PdO_*x*_) and the emergence of the carbide (PdC_*x*_) are accompanied by the short existence of a metallic surface.

**Figure 4:**
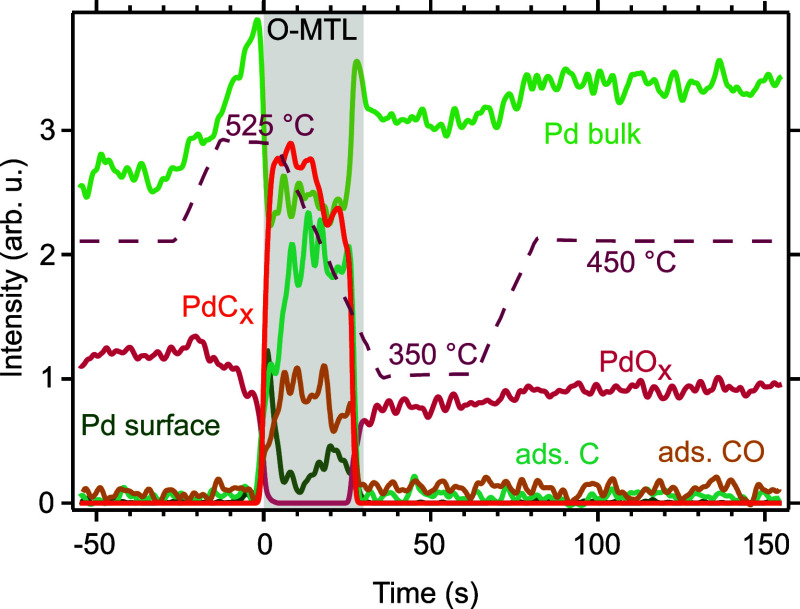
Smoothed fit results for the C 1s and Pd 3d_5/2_ surface spectra. The respective part of the catalyst temperature ramp is shown (dashed line).

The detailed analysis uses a fit to the data in Figure 3A,B, which results in BEs of the components, as shown in Table 2. The BEs for the surface spectral components are kept fixed and are based on the findings in refs (15) and (21), while the apparent C 1s BE of methane is allowed to vary by 0.4 eV (in agreement with the gas-phase analysis). The time development of the C 1s and Pd 3d_5/2_ fit intensities is presented in Figure 4. Since the fit results have a low signal-to-noise ratio, they were binomially smoothed 20 times in Figure 4, with the resulting errors shown in Figure S4. For the time axis, *t* = 0 was defined as the point in time at which the apparent C 1s BE of gaseous methane shifts by +0.4 eV. To synchronize the Pd 3d_5/2_ and C 1s data, common components were used (details on this can be found in the Supporting Information).

As was already qualitatively observed in Figure 3, it can now be observed in detail in Figure 4 that a carbide (PdC_*x*_ and ads. C) is detected on the surface during the whole O-MTL, i.e., *t* = 0 to *t* ≈ 30 s. To calculate the approximate thickness of this carbide, we performed the following analysis. The ratio between the intensities of Pd_bulk_ and PdC_*x*_ at *t* = 10 s in Figure 4 is roughly 0.9. To determine the thickness of the carbon layer, one can calculate the intensity ratio between Pd_bulk_ and PdC_*x*_ for an increasing number of carbon layers. The intensity of Pd_bulk_ can be calculated with the following formula
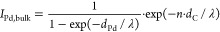
4where the first term is the limit of the geometric series, *d*_Pd_ the lattice constant of Pd, λ the mean free path of the electrons, *n* the number of carbon layers, and *d*_C_ the lattice constant of graphite. The same procedure can be applied for the intensity of PdC_*x*_
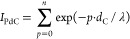
5

Calculating the ratio between these two values for *d*_Pd_ = 4 Å, *d*_PdC_ = 4.4 Å,^23^ and λ = 7 Å for electrons with a kinetic energy of 150 eV yields 2.30 for 1 ML of carbon and 0.80 for 2 MLs of carbon. Thus, the carbon coverage is slightly more than 1 ML. To calculate the approximate additional fraction of a ML of carbon atoms, a certain ratio *x* of the surface is assumed to be covered by 2 MLs of carbon while the remaining area is covered by 1 ML. Varying *x* to 0.9, i.e., 1.9 MLs of carbon coverage in total, leads to the desired ratio of 0.9 between the bulk Pd and the PdC_*x*_ component. A similar coverage is obtained when the attenuation of the Pd_bulk_ signal is used as the basis for the calculation (see Supporting Information). Even though these 1.9 ML are an approximate value, they are certainly not close to the 10^5^ ML as hinted upon in the gas-phase spectra. Additional to the carbon, adsorbed CO (ads. CO) detected from the C 1s curve-fitting is observed on the surface, having a similar time development as that of the gaseous CO component in the gas-phase data (see Figure 2). This leads to the conclusion that the local CO pressure determines the amount of adsorbed CO. Meanwhile, no oxide (PdO_*x*_) is observed at the surface. Outside of the O-MTL, however, the oxide is steadily growing, and the PdO_*x*_ intensity eventually reaches the same value as in the beginning of the presented time region, i.e., for *t* = −20 s (Figure S1). This was already indicated by the continuously changing sample work function observed in the discussion of the apparent BEs of the gas-phase components.

Focusing now on the component of adsorbed carbon, indications for carbon dissolution in the bulk can be found. In Figure 4, both the Pd 3d_5/2_ and the C 1s spectra show a sharp increase in this component (PdC_*x*_ and ads. C) at *t* = 0, i.e., when entering into the O-MTL, as well as a sharp drop when exiting the O-MTL at t ≈ 30 s. Here, the increase and the drop in surface carbon coverage occur abruptly between two consecutive measurement points in the unsmoothed data (see Figure S4), which points toward a surface development faster than the sampling rate, i.e., 0.2 s, as already indicated by the rapid changes in the apparent BE of the gas-phase components. Neither the time development of the ads. C nor the PdC_*x*_ component can be discussed in detail due to the high noise level in the fit results to the C 1s spectra (see Figure S4) and due to the fact that the PdC_*x*_ component includes both the time development of the adsorbed CO as well as that of adsorbed C binding to Pd atoms. It is, however, significant that both the ads. C and PdC_*x*_, components show a rather constant surface carbon coverage once they reach a certain value at the beginning of the O-MTL. This is surprising since we expect the continuous carbon deposition of approximately 10^5^ ML from the analysis of the gas-phase spectra. As these two observations do not agree with each other, carbon has to be removed from the surface, most likely by dissolution in the bulk. Additionally, a continuous net carbon deposition is suggested by the gas-phase spectra even shortly before and after the O-MTL. Since no carbon signal is measured in the topmost surface layers outside of the O-MTL, however, and no increased CO_2_ production is observed in the gas-phase spectra, which would be an indication for the oxidation of the carbide, carbon dissolution must also be present at these times. The carbon dissolution observed here agrees with the findings in a previous study, which, using thermogravimetry, could show that carbon deposition occurs with its subsequent removal from surface layers both by oxidation and by dissolution into the bulk.^24^

Relating the surface and gas-phase analyses, we observe that certain surface oxide phases limit methane adsorption. When looking at the end of the presented time region, the catalyst becomes almost inactive, as discussed in the analysis of the gas-phase spectra. The only development observed on the surface during this time is a growing surface oxide (PdO_*x*_). This suggests that certain surface oxide phases inhibit methane adsorption, which has been reported before for strongly adsorbed molecular oxygen and PdO patches.^19,25,26^

To summarize the findings reached by discussing the C 1s and Pd 3d_5/2_ surface spectra, we conclude that carbon is observed on the surface only within the O-MTL, even though we find evidence for carbon deposition already shortly before and after that. After exiting the O-MTL, growing oxide surface phases are observed. The surface developments deduced here agree with the literature, which found the formation of a subsurface carbon phase in a Pd catalyst during methane oxidation.^17,24,27^ Additionally, the observed rapid transition from an oxide, through a metallic surface, to a carbide phase is also supported by the literature.^19^

### Reaction Pathway Selectivity

**Figure 5:**
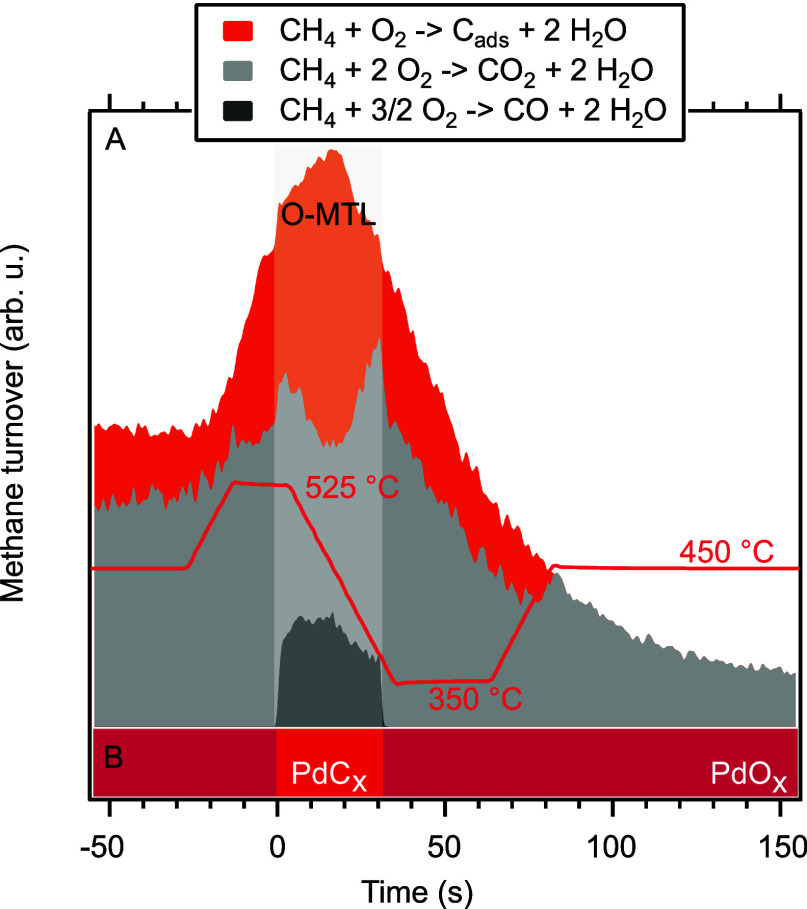
Deducted development of the selectivity toward all reaction pathways within the overall methane conversion over time in panel (A) together with the respective majority surface phase in panel (B).

Combining the analysis of the surface and gas-phase spectra, and using the water pressure as a measure for the (minimum) conversion rate, the amount of methane that is processed via each reaction channel (reaction eqs 1 to 3) can be calculated. Here, the remaining water signal after subtracting the complete 2*p*(CO_2_) and partial oxidation 2*p*(CO) contributions, according to eqs 2 and 3 must then be due to carbon deposition, i.e.,
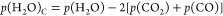
6since two water molecules are formed for each produced CO and CO_2_ molecule. The results for the selectivities toward each reaction pathway are shown in Figure 5A as a stacked plot within the (minimum) methane conversion, while panel B shows the majority surface phase over time.

A key observation in this figure is that, first of all, the selectivity toward individual reaction pathways changes within the O-MTL. That is, even though all three reaction channels are still operative, the increasing methane conversion leads to a shifting reaction pathway selectivity away from complete oxidation (CO_2_ formation) in favor of partial oxidation (CO production) and carbon deposition in the first half of the O-MTL. In the second half of the O-MTL, the reduced conversion rate then results in a back-shift of the reaction pathway selectivity. That is, no carbon is deposited at low conversion rates, while increasing conversion results in increased carbon deposition. Also, as opposed to, for instance, CO oxidation, the oxygen diffusion limitation does not limit the conversion rate. It even leads to an increasing conversion as the carbon deposition and CO formation channels become accessible for the conversion of methane. Outside of the O-MTL, complete oxidation is the dominating reaction pathway, however, as has been observed previously.^11^

Another striking observation is that we have a continuous net carbon deposition (approximately 10^5^ ML) during the O-MTL instead of just a spike, which would correspond to a filling of the surface layers with carbon. This is another indication of carbon dissolution in the bulk since the XPS surface signal of adsorbed carbon does not grow within the O-MTL. That means that the surface carbon coverage stays rather constant, which, in turn, means that the newly deposited carbon needs to be removed from the surface layers somehow. Additional evidence for carbon dissolution in the bulk comes from the fact that we observed topographical and color changes of the catalyst surface during the experiment (see Figure S7). The theory of carbon dissolution is further supported by mathematical modeling^18^ and thermogravimetry measurements.^24^

Additionally, an observation can be made that strongly supports the conclusion of carbon deposition outside of the O-MTL. When entering into the O-MTL between *t* = −10 s and *t* = 0, the carbon deposition signal (the orange area), which results from the excess water formation in the gas-phase spectra, increases continuously rather than stepwise. Even the entrance into the O-MTL happens smoothly from this point of view. Since we observe carbon on the surface in the O-MTL, i.e., the cause for the large carbon deposition signal in the O-MTL, carbon deposition must also be present just outside of the O-MTL. This indicates that carbon deposition increases with increasing conversion rates and temperatures, reaching its maximum in the middle of the O-MTL.

Relating the majority surface phase to the selected reaction pathway, it can be seen that partial oxidation to CO is only observed over carbide-covered surfaces, i.e., within the O-MTL, a behavior that has been observed previously.^12^ Interestingly, carbon deposition on the surface is observed not only within the O-MTL but also in a certain time frame around the O-MTL, i.e., on both oxide, metallic, and carbide surfaces. This is surprising since no carbon can be observed on the surface outside of the O-MTL. The reason for this is, most likely, that the diffusion rate of carbon into the bulk is faster than the carbon deposition rate; thus, no carbon surface phase can develop. Unfortunately though, no clear distinction could be found between the oxide surfaces that allow carbon deposition and those that do not. One explanation could be the existence of minority or short-lived metallic Pd sites, on which methane can adsorb and dissociate. Since no oxygen is available in the vicinity of the free carbon atom that dissolves in the bulk, leaving hydrogen to react with water, hence the excess water signal. The deposition on oxide surfaces, i.e., outside of the O-MTL, has not been observed previously since carbon deposition has so far been studied without focus on time resolution.^19,20,24^ Adsorbed carbon on the surface is then only observed during the O-MTL, since the carbon deposition rate increased drastically and is now larger than the diffusion rate. Toward the end of the presented time region, exclusively complete oxidation to CO_2_ is observed on oxide surfaces, however, only at fairly low conversion rates. The development of the reaction pathways over the entire temperature ramp, including the onset of carbon deposition on oxide surfaces, can be seen in Figure S5.

Concluding this discussion, we find that the O-MTL leads to a shift in the reaction pathway selectivity. Further evidence for carbon dissolution in the bulk and carbon deposition outside of the O-MTL was given. Furthermore, at low conversion rates, no carbon deposition is observed, while increased conversion results in carbon deposition.

## Conclusions

Methane oxidation over a polycrystalline Pd catalyst was studied with tr-APXPS in a methane-rich gas environment of 3.8 mbar. The spectra were measured continuously with a high time resolution (0.2 s) while applying a temperature ramp to the catalyst between 350 and 525 °C. The temperature-induced changes in the catalytic activity and, hence, gas-phase composition could be observed locally and in real time, together with the respective surface phases and their transitions.

**Figure 6:**
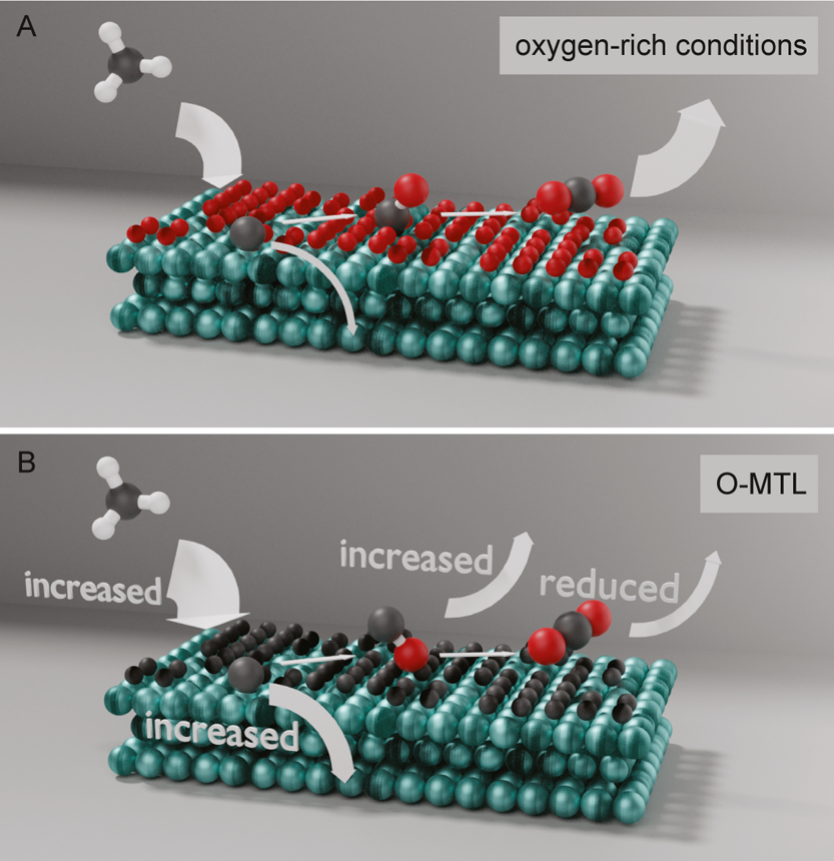
Schematic of reaction scheme for oxide (A) and carbide (B) Pd surface phases. The oxide phase develops in oxygen-rich conditions while the carbide phase is present during the O-MTL.

While studying this system, complete oxidation to CO_2_ is the dominating reaction pathway outside of the O-MTL, i.e., on oxide surfaces, as shown in panel A of Figure 6. Within the O-MTL, however, the selectivity toward each reaction pathway continuously changes. That is, an increasing partial oxidation to CO and carbon deposition together with a reduced complete oxidation to CO_2_ are observed on carbide surfaces, as is shown in panel B of Figure 6. On these surface phases, the maximum methane conversion is also reached, as observed before.^24,28^ Surprisingly, minor carbon deposition was also detected on oxide surfaces in a short time frame around the O-MTL (Figure 6A). We suggest that this happens on minority or short-lived metallic sites which coexist with the oxide. This is likely followed by carbon dissolution in the bulk.

Importantly, it is obvious from this study that local measurements of the gas composition are important for an understanding of catalytic function. For instance, we would have missed some of these results using time-resolved mass spectrometry measurements (see Figure S6) since it both lacks the ability to probe gas-phase products in the near vicinity of the surface as well as the ability to correctly measure the development of the water pressure. The latter was of crucial importance in this paper as a measure of the (minimum) conversion rate. Finally, the high time resolution in our measurements made it possible to observe that carbon deposition occurs outside of the O-MTL.

This study demonstrates how the water signal can be used as a measure of the conversion rate in hydrocarbon oxidation reactions. Together with the balanced reaction equations, this makes it possible to visualize previously hidden reaction channels, which was used in this study to observe how mass-transfer limitations can change the selected reaction pathway in systems with several possible reaction channels. We especially discovered that carbon deposition on the catalyst is not necessarily related to the observation of surface carbon coverage. In well-calibrated systems, this method can then even be used to quantitatively study carbon deposition. We expect this to have a high impact on the catalysis community since adsorbed or dissolved carbon can significantly modify catalytic performance.^1,4−8^

## Supporting Information Available

Experimental details, details on the data analysis, gas exchange time in the cell, calculations on carbon deposition and diffusion as well as thickness of the carbide layer, reproducibility of ramps, gas-phase and surface intensities (unsmoothed), selectivity during an entire ramp, QMS measurement, and evolution of the optical appearance of the sample (PDF)
