# Supplementary material for: Temperature-Dependent Selectivity and Detection of Hidden Carbon Deposition in Methane Oxidation
Source: ACS Catal. 2024 Apr 5;14(8):5978–86. doi: 10.1021/acscatal.4c00228 (PMC11036384; doi:10.1021/acscatal.4c00228)
Supplement: Supplementary file 1 — cs4c00228_si_001.pdf [file cs4c00228_si_001.pdf]

Supporting Information - Temperature-dependent selectivity and detection of hidden carbon deposition in methane oxidation

Ulrike Küst,[*,†,‡] Weijia Wang,[¶] Changda Wang,[§] Helena Hagelin-Weaver,[‖] Johan Gustafson,[†] Andrey Shavorskiy,[¶] Jason F. Weaver,[‖] and Jan Knudsen[*,†,¶,‡]

†*Division of Synchrotron Radiation Research, Lund University, Box 118, SE-221 00 Lund, Sweden*

‡*NanoLund, Lund University, Box 118, SE-221 00 Lund, Sweden*

¶*MAX IV Laboratory, Lund University, Box 118, SE-221 00 Lund, Sweden*

§*National Synchrotron Radiation Laboratory, University of Science and Technology of China, Hefei 230029, China*

‖*Department of Chemical Engineering, University of Florida, Gainesville, Florida 32611, United States*

E-mail: ulrike.kust@sljus.lu.se; jan.knudsen@sljus.lu.se

Experimental details

For these measurements a cylindrical sample was mounted on a transferrable 304L stainless steel sample plate. An IR laser in closed loop operation was used to heat the crystal and the temperature was monitored with a type K thermocouple spot welded to the side of the

crystals to ensure a precise temperature measurement. The reading was done once each second. To improve the signal-to-noise ratio in future experiments, averaging over several hundred of the temperature ramps applied here can be done. Thus, consecutive cycles can be used not only for a reproducibility check but also for improving the data quality.

Before the experiments the crystal was cleaned by 1 kV Ar^+ sputtering at $2{\cdot}10^{-5}$ mbar pressure and 10 mA emission current for 10 min followed by annealing to 700 °C. The cleanness of the surfaces was confirmed by XPS survey scans. The footprint of the beam on the sample is 60 × 25 μm and the measured gas phase signal originates from a volume consisting of this footprint size and a height of 90 μm. For all measurements at the HIPPIE beamline the R4000-HIPP-3 electron analyser was operated in fixed acquisition mode using a 5 Hz acquisition frequency. A large pass energy of 100 eV was used the surface spectra, while 200 eV was used during gas phase measurements such that the entire binding energy range (approximately 10% of the pass energy) could be covered by the electron analyzer in fixed acquisition mode. Instrument oxygen (5.0 N) and CH_4 (3.5 N) was used for the experiments. Commercial Pall gas cleaners (GLP2OXPVMM4 for O_2 and GLPSIPVMM4 for CH_4) were used on both gas lines. The gases were dosed with mass flow controllers (Brooks GF125). The stated flow values in sccm units refers to standard conditions of 20 °C and 14.696 psia (1 bar). The pressures stated in the paper were measured with an in-vacuum Pirani gauge (Pfeiffer-vacuum TTR 91).

To prepare the sample, a Pd(100) single crystal (6 mm diameter) was put through several oxidation and reduction cycles that roughened the surface, and, while changing color a few times (blue, light grey, green, black), the surface eventually turned polycrystalline. Survey spectra were collected throughout the measurement to check for possible contaminations. Impurities were not found at any point.

Hydrogen formation during methane oxidation has also been reported:[1]

$$CH_4 + \frac{1}{2}O_2 \longrightarrow CO + H_2. \tag{S1}$$

However, measuring hydrogen is not possible with XPS, which is why no conclusion can be drawn in this study related to the amount of produced hydrogen. That means, the water pressure is only a measure for the *minimum* methane conversion rate.

Data analysis

All data analysis was performed in Igor Pro 8 using purpose-written scripts. After calibration of the energy scale using the Fermi edge as a reference, polynomial background subtraction was carried out for each spectrum by fitting a polynomial to the datapoints where no components were visible. To determine the datapoint range for fitting the background subtraction the sum spectrum of all time resolved data was used (to ensure that the background subtraction did not remove any weak components). After background subtraction and normalization for varying electron transmission through the gas phase, all spectra were curve fitted with symmetric Voigt functions with as little free parameters as possible. For example, a common Lorentzian width was used for all components (0.1 eV[2]). In this work we did not correct for the analyzer transmission, which can affect the intensity. Neither the beamline flux nor the mean free path attenuation are evaluated since their effect is estimated to be small as compared to the noise observed in the raw data. Thus, the noise obtained from fitting the raw data is taken as a rough estimate of the error made.

When synchronizing the surface spectra, common components were used. That means that after defining t = 0 by means of the shift in the apparent binding energy of methane in the C 1s spectra, the carbide components in the C 1s and Pd $3d_{5/2}$ spectra (C_{ads} and PdC_x) were aligned. Finally, the oxide component in the Pd $3d_{5/2}$ and O 1s (PdO_x) spectra could be correlated.

Gas exchange time in the cell

To assess whether the changes observed in the gas phase spectra are influenced by the gas diffusion in the cell, the gas exchange time is calculated. Here, the gas cell has a volume of $V = 1$ l, the total gas flow is known to be $Q = 4$ sccm at a pressure of $p = 3.8$ mbar. Thus the exchange time is

$$t_{\text{ex}} = \frac{V}{Q_{1\text{ bar}}} = \frac{V}{Q} \cdot \frac{p}{1\text{ bar}} = 57s. \tag{S2}$$

This is ca. 10 times more than the time scales on which we measure but since we measure locally in the near vicinity of the sample surface, our results are most likely only marginally influenced by the gas exchange in the entire cell.

Carbon deposition and diffusion

When calculating the maximum number of monolayers of methane deposited during one second, the time it takes to form one monolayer is calculated first. The carbon that adsorbs on the surface must be equal to a methane partial pressure of 0.05 mbar (measured via the excess water signal) having a sticking coefficient of 1. According to kinetic gas theory this gives for the time to deposit one ML of carbon:

$$t = \frac{3 \cdot 10^{-4}\text{ Pa s}}{P \cdot 1\text{ML}} \tag{S3}$$

$$= \frac{3 \cdot 10^{-4}\text{Pa s}}{5\text{ Pa ML}} \tag{S4}$$

$$= 6 \cdot 10^{-5} \frac{\text{s}}{\text{ML}}. \tag{S5}$$

From this, the number of monolayers formed during one second is derived:

$$n_{\mathrm{ML}} = \frac{1}{6 \cdot 10^{-5}\ \mathrm{s/ML}} \tag{S6}$$

$$= 2 \cdot 10^4 \frac{\mathrm{ML}}{\mathrm{s}} \tag{S7}$$

For simplicity we consider a Pd(111) surface with $1 \cdot 10^{15}$ atoms/cm^2. This leads to a diffusion flux of:

$$J = 2 \cdot 10^4 \frac{\mathrm{ML}}{\mathrm{s}} \cdot 1 \cdot 10^{15} \frac{\mathrm{atoms}}{\mathrm{cm}^2} = 2 \cdot 10^{19} \frac{\mathrm{atoms}}{\mathrm{cm}^2 s} \tag{S8}$$

To estimate the theoretical carbon diffusion rate in Pd, we use a diffusivity of $D = 5 \cdot 10^{-7}$ cm^2/s.[3] The atomic density for Pd is $5 \cdot 10^{22}$ atoms/cm^3. At the surface (x=0) we assume a relative C to Pd concentration of 50 % while further into the crystal (x=0.1 cm), for simplicity we assume diffusion in the entire crystal, we assume a concentration of C atoms of 0 %. With Fick's law we get

$$J = -D \cdot \frac{dC}{dx} = 5 \cdot 10^{-7} \frac{\mathrm{cm}^2}{\mathrm{s}} \frac{0.5 \cdot 5 \cdot 10^{22} \mathrm{atoms/cm}^3}{0.1\mathrm{cm}} = 1.25 \cdot 10^{17} \frac{\mathrm{atoms}}{\mathrm{cm}^2\mathrm{s}} \tag{S9}$$

This is not on the same order of magnitude as the diffusion flux estimated from the measurement. If one, in the theoretical calculation, assumes diffusion within the topmost 10 μm, however, this is easily corrected.

Thickness of carbide layer

When calculating the number of monolayers of carbon present at the surface, two methods are possible. The first one (presented in the article itself) relies on the comparison between the Pd_{bulk} signal and the PdC_x signal during the existence of the carbide phase. Another one is based on the quantification of the attenuation of the Pd_{bulk} signal during the build-up

of the carbide phase. This approach is based on the assumption that the Pd surface is clean before the build-up of the carbide phase. Then, the Pd_{bulk} signal measured for a clean surface is

$$I_{\text{bulk at clean surface}} = I_0 \cdot \exp\left(-d_{\text{Pd}}/\lambda\right) \tag{S10}$$

where I_0 is the initial bulk signal before attenuation, d_{Pd} the lattice constant of surface Pd, and λ the mean free path of the escaping photoelectrons. For the Pd_{bulk} signal with a carbide coverage we get

$$I_{\text{bulk with carbide}} = I_0 \cdot \exp\left(-n d_{\text{PdC}}/\lambda\right). \tag{S11}$$

where d_{PdC} is the lattice constant of surface PdC and n the number of carbide layers. These two formulae can then be rewritten to provide the number of carbide layers:

$$n = \frac{1}{d_{\text{PdC}}} \cdot \left[d_{\text{Pd}} - \lambda \cdot \ln\left(\frac{I_{\text{bulk with carbide}}}{I_{\text{bulk at clean surface}}} \right) \right] \tag{S12}$$

For $I_{\text{bulk at clean surface}} = 3.9$, $I_{\text{bulk with carbide}} = 2.4$, and otherwise the same values as in the calculation in the article ($d_{\text{Pd}} = 4$ Å, $d_{\text{PdC}} = 4.4$ Å, $\lambda = 7$ Å), we get $n = 1.7$ which is reasonably close to the $n = 1.9$ obtained with the calculation in the article.

Figures

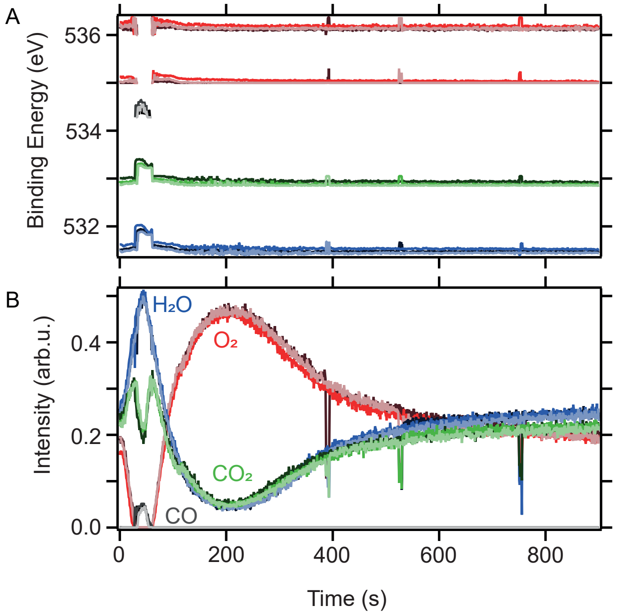

Figure S1: Proof of the reproducibility of the pulses and its effects on the XPS binding energies (A) and signal intensities (B) at the example of the O 1s and C 1s gas phase data. The brightest shade of the colors represents the first pulse while decreasing brightness accounts for the consecutive pulses. No binding energy is shown for components at zero intensity.

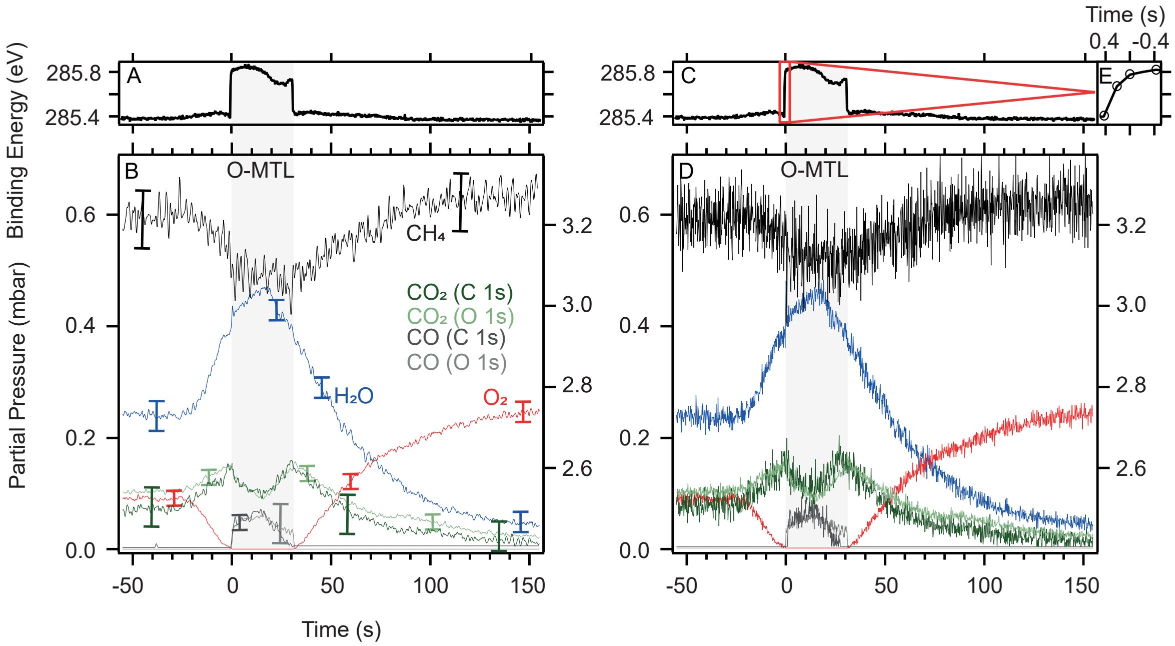

Figure S2: Apparent C 1s binding energy fit results for methane are shown (A). 75 times smoothed partial pressure fit results to the C 1s (CH_4, CO_2, CO) and the O 1s (H_2O, O_2, CO_2, CO) data are shown in Panel B together with the error bars resulting from the smoothing process. The methane component is accounted for by the right-hand axis while the other components refer to the left one. The same data are shown in Panels (C) and (D), respectively, before the smoothing process. The similarity in the curve shapes of the CO_2 and CO components measured in both the C 1s and O 1s core levels can be observed. Panel (E) shows an extract of the apparent C 1s binding energy of methane as indicated by the red box in (C) to illustrate the abrupt jump within two consecutive data points.

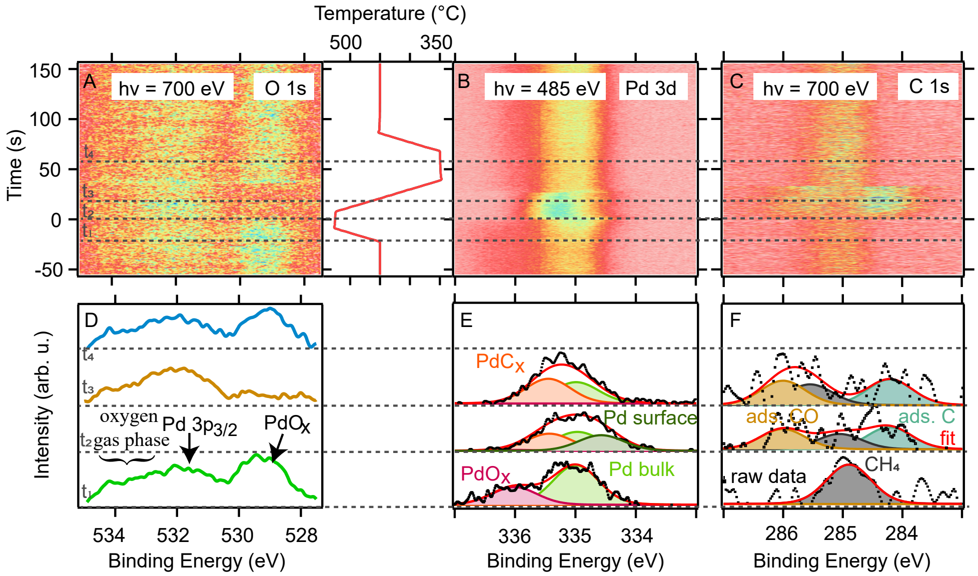

Figure S3: Measured raw data, i.e., the temperature of the catalyst, O 1s (A), Pd $3d_{5/2}$ (B), and C 1s (C) surface spectra. Examples of O 1s spectra, extracted and averaged over 70 spectra (D), and examples of fit to the raw data in Panels E and F.

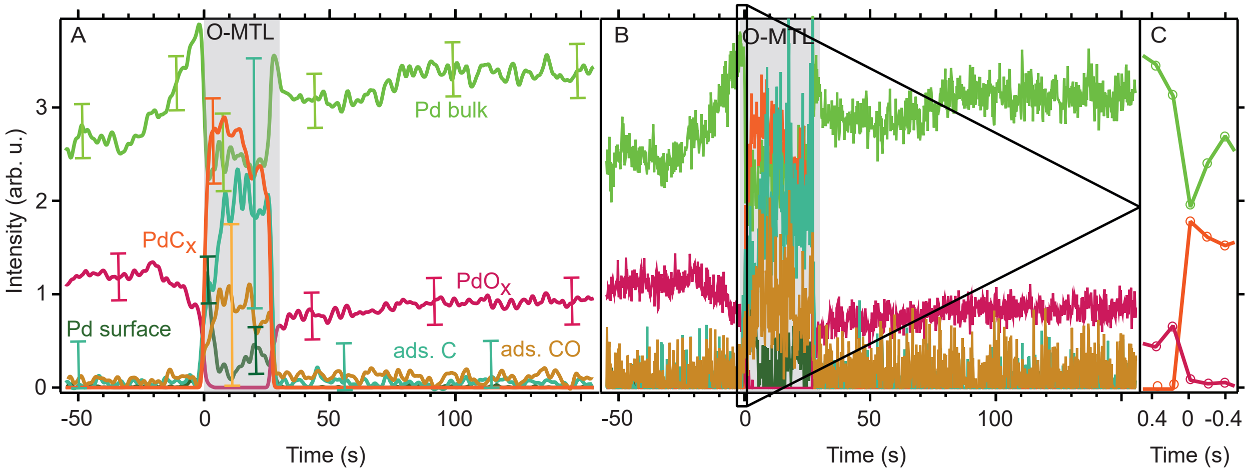

Figure S4: (A) Collocation of the 20 times smoothed fit results for the C 1s and Pd $3d_{5/2}$ surface spectra including the error bars that result from the smoothing process. (B) The same data before the smoothing process. (C) A zoomed-in image (shown by the black box in (B)) of the Pd bulk, the oxide, and the carbide component to visualize the jump within two consecutive data points.

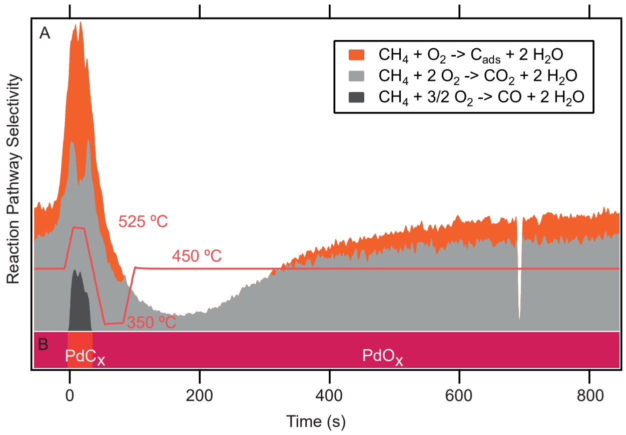

Figure S5: Deducted development of the selectivity towards all reaction pathways within the overall conversion over time in Panel A together with the respective majority surface phase in Panel B for an entire temperature ramp. Here, the end of the time axis is equivalent to its beginning, i.e., the development is periodic.

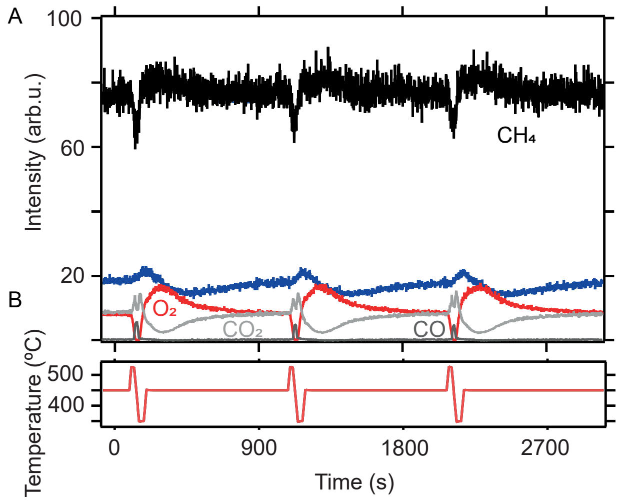

Figure S6: QMS data (A) following a temperature train (B). All gas phase components are visible, but measured centimeters away from the surface, thus, no direct conclusion of the local surface activity can be drawn. Especially the water signal is blurred due to the molecules sticking to the chamber walls. A detailed analysis of the QMS data is, hence, not possible.

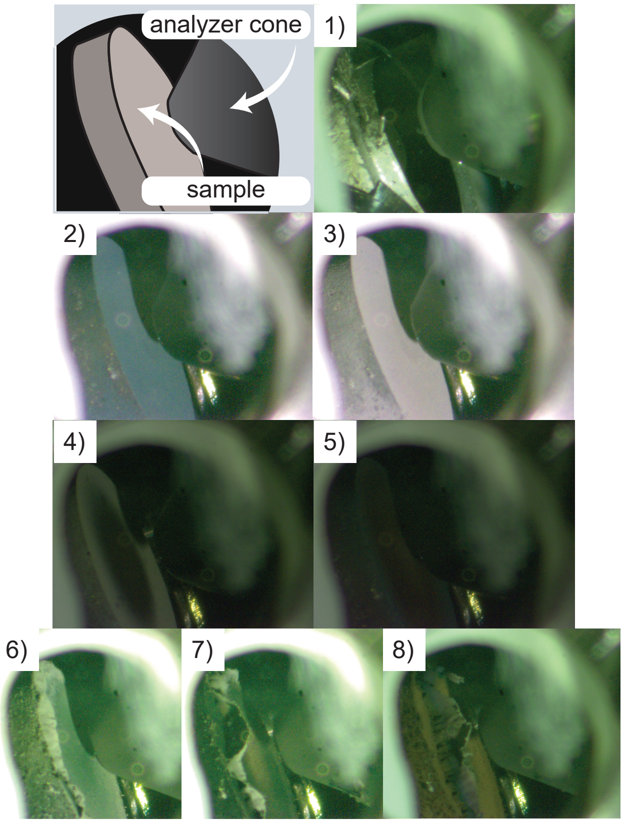

Figure S7: Starting out with a single crystal surface (1), it quickly turned white (2), then blue (3), and finally black (4,5). Eventually, the edges of the sample started producing flakes that slowly peeled of (6-8). During the entire time, however, the XPS survey showed nothing else than a metallic Pd signal (with oxygen and carbon). This is evidence for strong carbon dissolution in the bulk. The developing carbon layer has a different expansion as function of temperature than the metallic Pd lattice which leads to strain and the formation of wrinkles. This eventually results in the development of the observed flakes. The experiment presented in this article was performed on a surface phase close to the step 2.

References

(1) Diehm, C.; Deutschmann, O. Hydrogen production by catalytic partial oxidation of methane over staged Pd/Rh coated monoliths: Spatially resolved concentration and temperature profiles. 2014; pp 17998–18004.

(2) Campbell, J. L.; Papp, T. Widths of the atomic K-N7 levels. *Atomic Data and Nuclear Data Tables* **2001**, *77*, 1–56.

(3) Ling, C.; Sholl, D. S. First-principles evaluation of carbon diffusion in Pd and Pd-based alloys. *Physical Review B - Condensed Matter and Materials Physics* **2009**, *80*.
